# Lack of impact of radiation on blood physiology biomarkers of Chernobyl tree frogs

**DOI:** 10.1186/s12983-021-00416-x

**Published:** 2021-06-29

**Authors:** Pablo Burraco, Jean-Marc Bonzom, Clément Car, Karine Beaugelin-Seiller, Sergey Gashchak, Germán Orizaola

**Affiliations:** 1 Institute of Biodiversity, Animal Health and Comparative Medicine, College of Medical, Veterinary and Life Sciences, University of Glasgow, G12 8QQ, Glasgow, UK; 2 Animal Ecology, Department of Ecology and Genetics, Evolutionary Biology Centre, Uppsala University, 75236 Uppsala, Sweden; 3 Institut de Radioprotection et de Sûreté Nucléaire (IRSN), PSE-ENV/SRTE/LECO, Cadarache, 13115 Saint Paul Lez Durance, France; 4 Chornobyl Center for Nuclear Safety, Radioactive Waste and Radioecology, Slavutych, 07100 Ukraine; 5 IMIB-Biodiversity Research Institute (Univ. Oviedo-CSIC-Princip. Asturias), University of Oviedo, 33600 Mieres, Asturias Spain; 6 Zoology Unit, Department of Biology of Organisms and Systems, University of Oviedo, 33071 Oviedo, Asturias Spain

**Keywords:** Amphibians, Biochemical blood parameters, Ionizing radiation, Vertebrate physiology

## Abstract

**Background:**

Human actions have altered natural ecosystems worldwide. Among the many pollutants released to the environment, ionizing radiation can cause severe damage at different molecular and functional levels. The accident in the Chernobyl Nuclear Power Plant (1986) caused the largest release of ionizing radiation to the environment in human history. Here, we examined the impact of the current exposure to ionizing radiation on blood physiology biomarkers of adult males of the Eastern tree frog (*Hyla orientalis*) inhabiting within and outside the Chernobyl Exclusion Zone. We measured the levels of eight blood parameters (sodium, potassium, chloride, ionized calcium, total carbon dioxide, glucose, urea nitrogen, and anion gap), physiological markers of homeostasis, as well as of liver and kidney function.

**Results:**

Levels of blood physiology biomarkers did not vary in function of the current exposure of tree frogs to ionizing radiation within the Chernobyl Exclusion Zone. Physiological blood levels were similar in frogs inhabiting Chernobyl (both in areas with medium-high or low radiation) than in tree frogs living outside Chernobyl exposed only to background radiation levels.

**Conclusions:**

The observed lack of effects of current radiation levels on blood biomarkers can be a consequence of the low levels of radiation currently experienced by Chernobyl tree frogs, but also to the fact that our sampling was restricted to active breeding males, i.e. potentially healthy adult individuals. Despite the clear absence of effects of current radiation levels on physiological blood parameters in tree frogs, more research covering different life stages and ecological scenarios is still needed to clarify the impact of ionizing radiation on the physiology, ecology, and dynamics of wildlife inhabiting radioactive-contaminated areas.

**Supplementary Information:**

The online version contains supplementary material available at 10.1186/s12983-021-00416-x.

## Background

Recent human activity has caused abrupt environmental change in the form of habitat destruction and fragmentation, the spread of exotic species, or the severe alteration of climatic conditions [1]. Human action may also include the release of novel substances to the environment, with the potential to affect the behavior, life-history, and/or physiology of organisms, which can have important consequences on fitness [2]. The exposure to pollutants, in particular, can induce many biochemical and physiological alterations in wildlife [3, 4]. The magnitude of such effects often depends on the nature and concentration of the pollutant, the duration of the exposure, and the ecology and evolutionary history of the species [4, 5].

All organisms are constantly exposed to very low levels of ionizing radiation, coming mostly from cosmic rays and naturally occurring radioactive materials (i.e. background ionizing radiation [6];). However, human activity has caused the accidental release of vast amounts of ionizing radiation to the environment, as on the accidents on the nuclear power plants of Chernobyl (Ukraine, 1986) and Fukushima (Japan, 2011). The acute exposure to ionizing radiation can induce severe health problems in vertebrates. Ionizing radiation is known to damage organic molecules, disrupt physiological processes such as the redox status, and cause chronic inflammatory responses or cell apoptosis [7–11]. Although the effects of an acute exposure to ionizing radiation are clear, there is still intense scientific debate about the mid- and long-term effects that the chronic exposure to radiation has in wildlife living on radio-contaminated areas (e.g. [8, 12, 13]).

The accident at the Chernobyl Nuclear Power Plant, on 26th April 1986, represents the largest release of ionizing radiation in human history. Radiation levels in areas near the power plant increased up to one million times after the accident [14]. As a consequence of the accident, an exclusion zone of ca. 4700 km^2^ was created in Ukraine and Belarus, and public access and inhabitation were restricted. This situation provides a unique scenario for the study of the ecological and evolutionary consequences of the chronic exposure to ionizing radiation in wildlife [13, 15]. The immediate effects of the Chernobyl radioactive fallout in wildlife were severe [8, 14, 16]. However, more than three decades have passed since the accident, and radiation levels in the area have decreased several orders of magnitude [17]. Recent studies have reported the increase of mammal densities [18] and the arrival to the Chernobyl area of species not present at the time of the accident (brown bear [19], European bison [20]). Other studies have even suggested that chronic exposure to radiation may have favored adaptive responses to cope with current radiation levels (e.g. [21, 22]; but see [12]). On the other hand, exposure to radiation has been still associated with negative effects on the physiology of several animal species (insects [23]:; birds [24, 25]; mammals [26]:). For example, radiation has detrimental consequences for the redox status and for DNA stability in the barn swallow *Hirundo rustica* [11, 27, 28]. However, these effects might be taxa-dependent, as suggested by the lack of effects observed in the offspring of grasshoppers exposed to radiation [29].

Amphibians are ideal subjects to study the effects of ionizing radiation on vertebrates. The life cycle of amphibians is often biphasic, including stages in water (embryonic and larval) and on land (juvenile and adult), so individuals are exposed throughout their life to radiation coming from aquatic and terrestrial sources. Even more relevant, they have a reduced dispersal capacity and high philopatry [30], allowing a precise evaluation of their exposure to environmental radiation. Few studies have examined the effects of the exposure to ionizing radiation in wild amphibians, and even fewer ones have tried to understand its effect on their physiology [31]. Recent studies on the Japanese tree frog (*Hyla japonica*) in Fukushima have reported, for example, no effects of dose rates on carotenoid levels in blood, liver, or vocal sac, but a dose-dependent increase in DNA methylation and mitochondrial DNA damage [32, 33]. The examination of blood physiology parameters has been extensively used for determining human health (e.g. [34]), as well as in wildlife exposed to differences sources of environmental stress (e.g. [35, 36]). The use of physiological blood parameters in the evaluation of animal health is supported by the link between electrolyte levels and body homeostasis, and by the role that some of these parameters have as indicators of liver and kidney malfunction [37]. Alterations in blood electrolytes have been reported in vertebrates exposed to ionizing radiation under laboratory conditions (e.g. [38]) and are common in cancer patients exposed to radiotherapy (e.g. [39]). The development of portable point-of-care devices has simplified the evaluation of blood electrolytes under field conditions, and therefore has become a powerful tool to determine the health of wildlife [40–42].

Here, we examined the impact of the current exposure to ionizing radiation on blood physiology biomarkers of amphibians. In amphibians, the maintenance of blood electrolyte levels is key for body homeostasis and, more specifically, for the correct function of kidney and liver, as well as for skin health [35, 43, 44]. In this study, we used a portable point-of-care device to quantify different biochemical blood parameters [42] (see details below) in adult males of the Eastern tree frog (*Hyla orientalis*) inhabiting within and outside the Chernobyl Exclusion Zone, Ukraine. In particular, we examined blood physiology biomarkers in relation to the total dose rate of radiation absorbed by each frog, an approach that allows to accurately address the effect of radiation at the individual level [45]. We also compared physiological blood levels of frogs inhabiting within Chernobyl (both in medium-high- and low-radiation areas) and outside the Chernobyl area, in order to examine the possible effects that divergent historical and current exposure to radiation might represent for amphibian health. Considering previous studies on the physiological effects of ionizing radiation on wildlife (e.g. [27, 28]), we hypothesized that the chronic exposure to high radiation levels (i.e. individuals with higher dose rates) will negatively impact the blood physiology of frogs. We also hypothesized that frogs inhabiting within high radiation areas would experience imbalanced blood physiology compared to frogs from low radiation and control areas in the Chernobyl Exclusion Zone. However, the reduction in radiation levels after three decades from the accident plus the strong selection processes that likely have already occurred within Chernobyl, may result in a lack of impact of ionizing radiation on the biomarkers of blood physiology of breeding frogs.

## Results

All individuals survived from collection until they were sacrificed for blood physiology assessments. Mean body length (snout-to-vent length, SVL) of *H. orientalis* males used in the study was 38.92 ± 0.49 mm, and mean body mass 5.43 ± 0.16 g (see Supplementary Data). Activity of radionuclides in *H. orientalis* male frogs collected within Chernobyl Exclusion Zone ranged from 0 to 25.1 Bq/g for ^137^Cs and from 0.2 to 248.9 Bq/g for ^90^Sr, and total individual dose rates ranged from 0 to 36.28 μGy/h (see Supplementary Data; Figure S1). Values for individuals collected outside Chernobyl Exclusion Zone were always below detection levels.

**Fig. 1:**
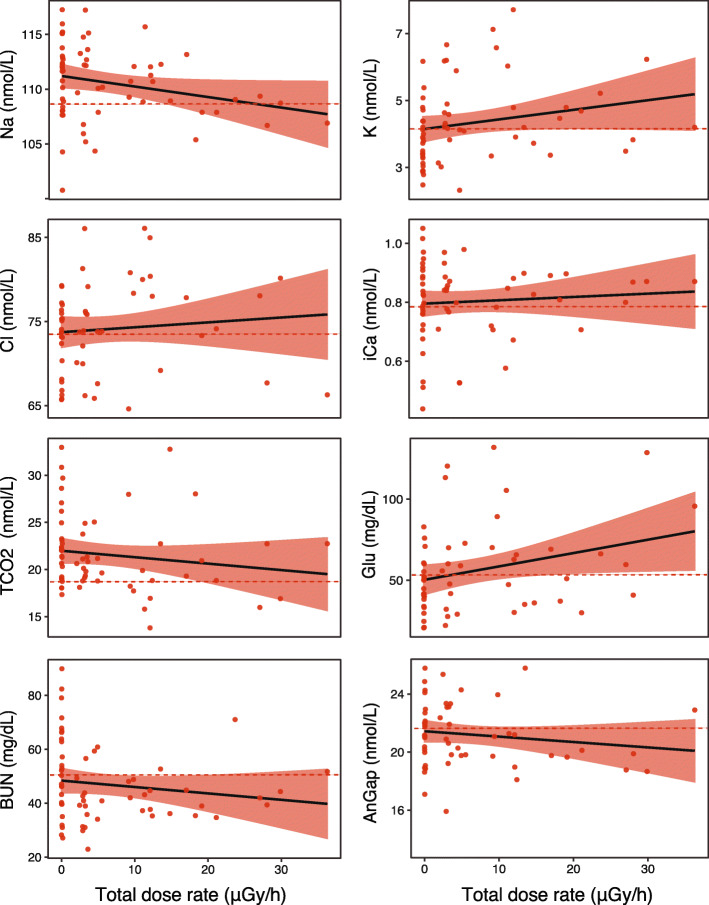
Regressions between total individual dose rates (in μGy/h) and levels of the examined physiological blood parameters in adult males of the Eastern tree frog (*Hyla orientalis*) inhabiting within the Chernobyl Exclusion Zone. See text for parameter codes. Red bands represent 95% confidence intervals. The dashed red line indicates the mean value of each parameter for control frogs, i.e. those from locations outside Chernobyl (reference values for this study)

**Fig. 2:**
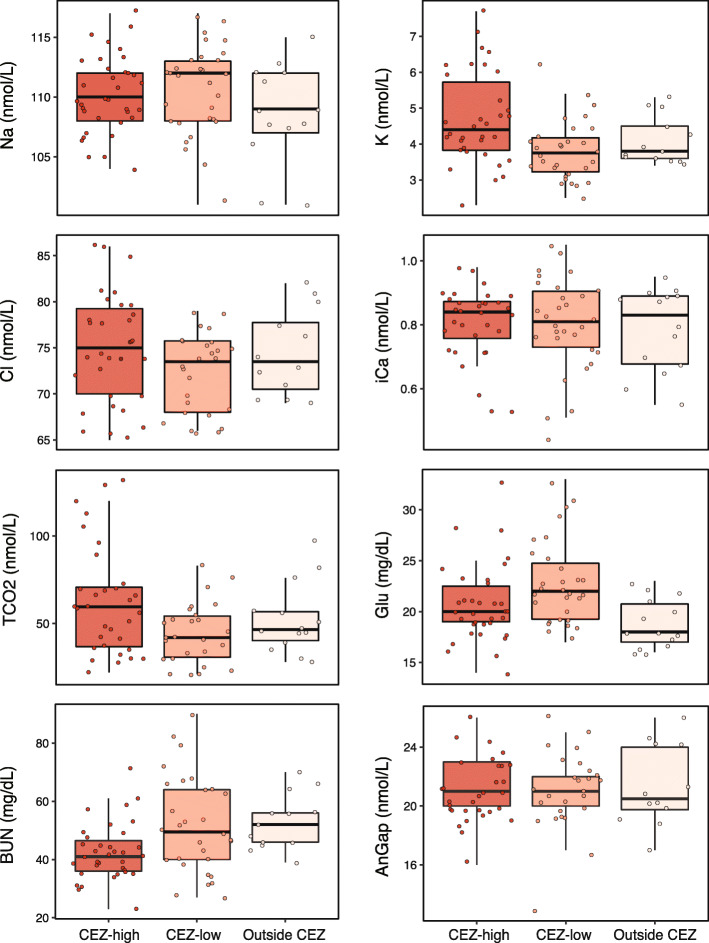
Levels of physiological blood parameters of adult males of the Eastern tree frog (*Hyla orientalis*) collected in areas of medium-high radiation (CEZ-High), and low radiation (CEZ-low) within the Chernobyl Exclusion Zone, and areas outside the Chernobyl Exclusion Zone (Outside CEZ). See text for parameter codes. Box plots represent the interval between first and third quartiles, black lines depict the median value, bars represent minimum and maximum values within 1.5 times interquartile range

Current exposure levels to ionizing radiation (i.e. total individual dose rates) of *H. orientalis* males inhabiting within Chernobyl Exclusion Zone had no effect on the levels of any of the physiological blood parameters examined (*P* > 0.078 in all cases; Table 1; Fig. 1). Body condition index did not affect physiological parameters (except for urea nitrogen, BUN, *P* = 0.048; Table 1). We found no differences in the levels of any *H. orientalis* blood parameter between male frogs inhabiting Chernobyl Exclusion Zone (neither high- or low- radiation areas) and male tree frogs from Outside Chernobyl, exposed only to background radiation levels (*P* > 0.06 in all cases; Table 2; Fig. 2).

**Table 1:** Effects of total individual dose rate and body condition index on physiological blood parameters of adult breeding Eastern tree frog (*Hyla orientalis*) males inhabiting within the Chernobyl Exclusion Zone. Abbreviations are: sodium (Na), potassium (K), chloride (Cl), ionized calcium (iCa), total carbon dioxide (TCO_2_), glucose (Glu), urea nitrogen (BUN), and anion gap (AnGap)

	Total individual dose rate			Body condition index		
	Chi-sq	Estimate	*P*-value	Chi-sq	Estimate	*P*-value
**Na**	0.57	−0.006	0.450	1.98	1.979	0.159
**K**	3.11	3.114	0.078	0.65	0.648	0.421
**Cl**	0.002	0.002	0.963	0.22	0.219	0.640
**iCa**	0.14	0.136	0.712	0.002	0.002	0.967
**TCO**_**2**_	0.83	0.835	0.361	0.02	0.021	0.883
**Glu**	2.95	2.947	0.086	0.92	0.922	0.337
**BUN**	0.0002	0.0002	0.987	3.91	3.906	0.048
**AnGap**	0.29	0.298	0.585	0.01	0.011	0.916

**Table 2:** Levels of physiological blood parameters, and effects of environmental radiation category on adult breeding Eastern tree frog (*Hyla orientalis*) males collected in areas of medium-high radiation (CEZ-High, *n* = 35), and low radiation (CEZ-Low, *n* = 30) within the Chernobyl Exclusion Zone, and areas outside the Chernobyl Exclusion Zone (Outside CEZ, *n* = 14). Blood parameters were: sodium (Na), potassium (K), chloride (Cl), ionized calcium (iCa), total carbon dioxide (TCO_2_) and anion gap (AnGap), all in mmol/L; and glucose (Glu), and urea nitrogen (BUN), both in mg/dL. Physiological values are presented as least square means ± SE, including the range of values, and sample size for each category

	Physiological blood levels						*P*-value
	CEZ-High		CEZ-Low		Outside CEZ		
**Na**	111.0 ± 1.1	(117–104; *N* = 35)	110.0 ± 1.1	(117–101; *N* = 30)	108.0 ± 1.4	(115–101; *N* = 13)	0.278
**K**	4.6 ± 0.3	(7.7–2.3; *N* = 34)	3.9 ± 0.3	(6.2–2.5; *N* = 30)	4.1 ± 0.4	(5.3–3.4; *N* = 13)	0.134
**Cl**	74.5 ± 2.4	(86–65; *N* = 32)	72.4 ± 2.5	(79–66; *N* = 26)	73.9 ± 3.1	(82–69; *N* = 12)	0.750
**iCa**	0.8 ± 0.1	(0.98–0.53; *N* = 32)	0.8 ± 0.1	(1.05–0.44; *N* = 30)	0.8 ± 0.1	(0.95–0.55; *N* = 14)	0.917
**TCO2**	21.0 ± 1.7	(33–14; *N* = 34)	23.0 ± 1.7	(33–17; *N* = 30)	18.7 ± 2.1	(23–16; *N* = 14)	0.190
**Glu**	64.2 ± 7.0	(132–22; *N* = 32)	43.8 ± 7.4	(83–21; *N* = 26)	52.5 ± 9.0	(97–28; *N* = 14)	0.066
**BUN**	44.5 ± 4.1	(71–23; *N* = 35)	49.9 ± 4.2	(90–27; *N* = 30)	52.4 ± 5.4	(70–39; *N* = 13)	0.356
**AnGap**	21.1 ± 0.8	(26–16; *N* = 31)	20.8 ± 0.8	(26–13; *N* = 26)	21.5 ± 1.0	(26–17; *N* = 12)	0.904

## Discussion

Our study reveals that, more than thirty years after the nuclear accident, radiation levels currently experienced by breeding males of the Eastern tree frog (*Hyla orientalis*) had no effect on several physiological blood parameters, surrogates of individual health (i.e. body homeostasis, liver and kidney damage, skin health). Furthermore, frogs inhabiting within the Chernobyl Exclusion Zone, both in high- and low-radiation areas, had similar physiological blood levels than those from locations outside the Chernobyl area experiencing only background levels of radiation.

The immediate negative consequences for health of the acute radiation levels generated by the Chernobyl accident have been broadly reported both in humans [46–48] and wildlife (reviewed in [49] [8, 14, 16];). However, current exposure to low or moderate radiation levels seems to impair vertebrate physiology in a very variable way, likely dependent on ecological and demographic contexts, even within the same taxa. For example, while some studies have reported negative effects of ionizing radiation on parameters indicative of oxidative stress levels and DNA damage in birds [27], other studies have shown that exposure to radiation might be favoring adaptive processes in some bird species inhabiting Chernobyl [22]. Here, we found no relation between a very accurate measure of individual exposure to radiation (i.e. individual dose rate [45];) and levels of several physiological blood parameters, which are widely used in clinical and wildlife veterinary as markers of homeostasis, and of kidney and liver damage [41, 50, 51], and affected by ionizing radiation [38].

Values for the studied blood biomarkers were rather similar to those reported in previous studies with amphibians in uncontaminated environments (e.g. [35, 52, 53]). Different factors can help to place this output into context. First, due to radionuclide decay and the short life of many of the released radionuclides (i.e. ^131^I), current radiation levels represent ca. 10% of the levels at the time of the accident [54], and may not be high enough as to cause the type of physiological imbalances addressed by these biomarkers. Actually, although frogs were collected in breeding areas located across the current gradient of radioactive contamination for the species in Chernobyl, many of the examined individuals had dose rates lower than 10 μGy/h, which is the dose rate below which negative effects are not expected, based on international reference values [55]. These reference values, however, should be considered carefully, since they can be rather arbitrary (see e.g. [56]) and are often not based in field assessments.

Another relevant point is that our study focused on active males, calling at night in breeding ponds, which would be considered on itself as an indication of good health. On the contrary, if some individuals are affected by radiation, they may shift energy investment from reproduction to somatic maintenance, and thus go unnoticed. Ionizing radiation is also known to affect organisms more severely at early developmental stages (i.e. embryos or young individuals; e.g. [57, 58]), therefore selection driven by chronic radiation may have purged already less resilient individuals. This can be particularly important for species with complex life cycles like amphibians, as their sensitiveness to radiation might be different at pre-metamorphosis or adult stages [31]. Alternatively, exposure to radiation during three decades may have acted as a strong selective force, removing vulnerable individuals and favoring individuals more capable to adjust their physiology in order to avoid costs of radiation exposure, hence leading to a lack of variation among areas in the examined blood parameters.

## Conclusions

Current exposure to ionizing radiation does not to affect levels of blood physiology biomarkers (used as proxies of general physiological imbalance, or kidney and liver damage) in Eastern tree frogs (*Hyla orientalis*) breeding males inhabiting within and outside the Chernobyl Exclusion Zone. However, more research including different species, life stages, and eco-evolutionary scenarios is needed for a better understanding of the effects that ionizing radiation has on wildlife physiology and specifically on amphibians. Future studies should ideally also include laboratory tests with low-dose radiation, as well as field experiments, to disentangle the eco-evolutionary consequences of radioactive contaminated environments.

## Methods

**Fig. 3:**
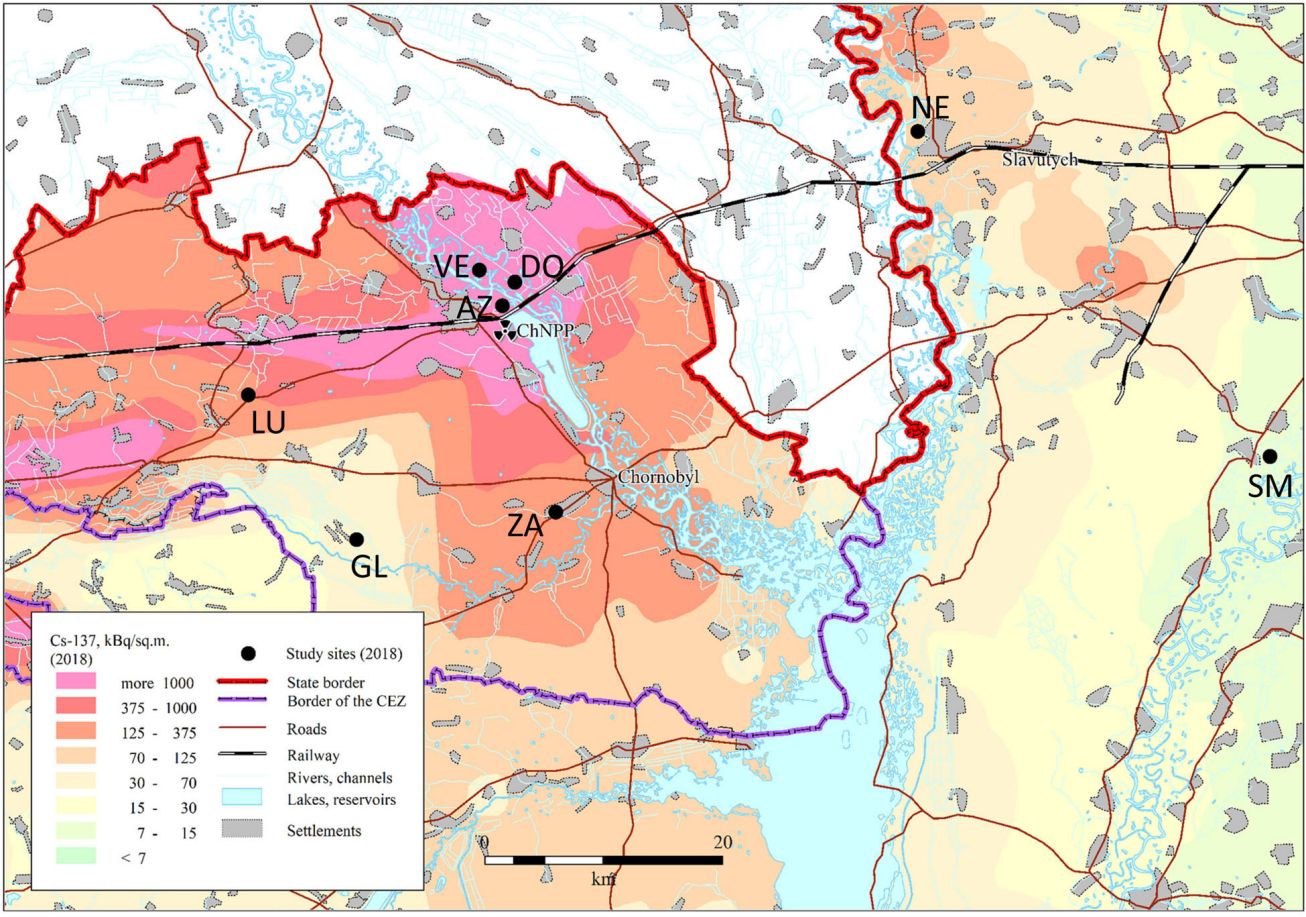
Map showing the studied Eastern tree frog (*Hyla orientalis*) locations. The abbreviations refer to the location name. Azbuchin (AZ), Vershina (VE), Dolzhikovo (DO), located in high radiation areas within the Chernobyl Exclusion Zone; Zalesie (ZA), Lubianka (LU), and Glinka (GL), located in low radiation areas within the Chernobyl Exclusion Zone; and Smolin (SM) and Nedanchichy (NE), located outside Chernobyl (see Table S1 for details). The underlying ^137^Cs soil data (decay corrected to spring 2018) is derived from the Atlas of Radioactive Contamination of Ukraine (Intelligence Systems GEO, 2011)

The Eastern tree frog (*Hyla orientalis*) is a cryptic species of the European tree frog (*Hyla arborea*) group that inhabits eastern Europe, Anatolia, and the Caucasus [59]. On May 2018, we collected 101 adult *H. orientalis* males at eight locations in Northern Ukraine (Fig. 3, Supplementary Material Table S1). Six of these locations are within the Chernobyl Exclusion Zone (CEZ), three in areas with medium-high radiation levels (range: 16–20-1.50 μSv/h; thereafter *CEZ-High*) and three in areas with low radiation (range: 0.27–0.10 μSv/h; thereafter *CEZ-Low*; Supplementary Material Table S1). Two additional locations are outside the Chernobyl Exclusion Zone (~ 40 km East, Fig. 3), in areas that maintain background radiation levels (i.e. ≤ 0.08 μSv/h; thereafter called *Outside CEZ*; Supplementary Material Table S1). We captured active calling frogs during the night from 9 PM to 12 PM. Temperature during the sampling nights were very similar between areas (average: 14.6 ± 1.2 °C inside Chernobyl; 14.2 ± 0.5 °C outside Chernobyl). All the collected frogs appeared clinically healthy. Once captured, frogs were transported to the laboratory, placed individually in plastic boxes with 50 mL of clean water, and kept overnight at a temperature ranging between 19 and 21 °C. On the following morning, we measured body length (snout-vent length, SVL), width, and height of each frog to the nearest 1 mm with the help of a caliper, and body mass on a balance to the nearest 0.01 g. These morphometric measurements were used to estimate individual dose rates and body condition (see below).

### Blood physiology

We examined blood physiology of frogs using the VetScan i-STAT portable clinical analyzer for point-of-care blood testing, and i-STAT CHEM 8+ test cartridges (Abaxis, Union City, CA, USA). This blood analyzer gives accurate measurements of biochemical blood parameters and has been extensively tested in wild vertebrates (e.g. [60–62]; reviewed in [42]). The CHEM 8+ test cartridge gives estimates of sodium (Na), potassium (K), chloride (Cl), ionized calcium (iCa), total carbon dioxide (TCO_2_), glucose (Glu), urea nitrogen (BUN), creatinine (Crea), and hematocrit (Hct), and calculates the concentration of anion gap (AnGap) and hemoglobin (Hgb), parameters linked to homeostasis maintenance and health in amphibians [35, 43, 44]. Sodium, potassium, chloride, and ionized calcium are essential for electrolyte and fluid balance; glucose is the principal energy source in animals; urea is an indicator of liver function; and anion gap represents the balance between cations and anions in blood [42, 50, 51]. All cartridges were stored at 4 °C until use and assayed at room temperature to avoid any temperature effect on the measurements (e.g. [63]).

Once morphometric measurements were recorded, we euthanized frogs by pithing without decapitation [64], and collected blood on 100 μL heparinized capillary tubes. We quickly filled the CHEM 8+ cartridge with ca. 95 μL of blood and ran the tests. For some individuals we were not able to collect the required amount of blood, whereas in other cases the portable point-of-care device reported error messages, mostly caused by the presence of air bubbles in the sample. We discarded two parameters, creatinine and hematocrit, because the estimated concentration was below the detectable limit of the blood analyzer in many individuals (56.96 and 36.7% of creatinine and hematocrit measurements, respectively). Since i-STAT analyzer calculates hemoglobin concentration from hematocrit values, we also discarded hemoglobin from further analyses. Overall, we used in our analyses eight physiological blood parameters measured in 79 frogs: 35 from locations with medium-high radiation levels within Chernobyl Exclusion Zone (*CEZ-high*), 30 from locations in low radiation areas within the Exclusion Zone (*CEZ-low*), and 14 from locations outside Chernobyl (*Outside CEZ*; Fig. 3). In order to estimate intra-sample variation and technical replicability, we ran ca. 15% of samples in duplicate (*n* = 11), using two different cartridges per individual. The coefficients of variation for these duplicated samples were: sodium (0.64%), potassium (11.41%), chloride (2.18%), ionized calcium (6.81%), total carbon dioxide (2.81%), glucose (10.41%), urea nitrogen (6.32%), and anion gap (11.91%).

Since there is no precise information on baseline levels for these parameters in *Hyla orientalis*, we used the levels of individuals from locations outside the Exclusion Zone (*Outside CEZ*) as reference values.

### Radiological evaluation

We estimated ambient dose rate using a MKS-AT6130 radiometer placed at 5 cm above the surface of the pond shoreline (5 measurements per pond, see [65] for details). The method used to estimate total individual dose rates absorbed by each frog during the breeding period (in μGy/h) is described in detail in [32] with the few minor modifications described below. We estimated soil activities (in Bq/Kg) for ^137^Cs and ^90^Sr (the dominant radionuclides in Chernobyl [66];) in the studied locations using a spatial database derived from the integration of the airborne gamma survey with results of soil sampling in earlier 1990s ( [65] for details). Water activities (in Bq/L) were calculated using soil activities data and distribution coefficients estimated for the Glubokoye lake in Chernobyl [67]. We measured radionuclide activity concentrations at the Chornobyl Center for Nuclear Safety, Radioactive Waste and Radioecology laboratories using beta spectrometry in femur bones for ^90^Sr (β-spectrometer EXPRESS-01), and gamma spectrometry in leg muscle for ^137^Cs (Canberra-Packard gamma-spectrometer with high-purity germanium detector GC 3019; see [65] for details). We estimated the total activity in ^90^Sr and ^137^Cs for each frog, by integrating the radionuclide measurements with the body mass value of each individual and considering the relative mass of bones (10%) and muscles (69%). Briefly, in order to estimate total individual dose rates, we combined radionuclide activity concentrations in frogs, in soil, in water, and dose coefficients (in μGy/h per Bq per unit of mass). Dose coefficients for *H. orientalis* were calculated for internal exposure, and for external exposure considering a single scenario that integrates four ecologically situations that are experienced by tree frogs across a whole breeding season [32, 65] using EDEN v3 IRSN software [68]. For each frog, the total individual dose rate was therefore calculated by summing internal and external dose rates.

### Statistical analyses

We ran all statistical analyses in R, version 3.6.1 (R Development Core Team). We checked for parametric assumptions by running Kolmogorov-Smirnov tests for normality data assessments (*lillie.test* function, included in the package *nortest*, version 1.0–2) and Breusch-Pagan tests for homoscedasticity data assessments (*bptest* function, included in the *lmtest* package, version 0.9–35). Data of all blood parameters and total dose rate (once added 0.1 unit to each value) were log-transformed to meet parametric assumptions. To check for the effect of individual dose rate on blood parameters in frogs inhabiting the Chernobyl Exclusion Zone, we conducted linear mixed models including each blood parameter as dependent variable, total individual absorbed dose rate as predictor variable, body condition index as covariate, and location as random factor (package *lme4*, version 1.1–23; Supplementary Material). Body condition was estimated as the residuals obtained from the linear regression between mass and length [69]. We also investigated the effect of environmental radiation category (i.e. *Chernobyl Exclusion Zone* or *Outside Chernobyl*) on the blood physiology of frogs, by running linear mixed models including each blood parameter as dependent variable, environmental radiation category as independent variable, body condition index as covariate, and sampling location as random factor (package *lme4*, version 1.1–23). From each linear mixed model, estimated marginal means were calculated using the function *emmeans* (version 1.5.3). Data were plotted using the package *ggplot2* (version 3.3.0).

## Supplementary Information

**Additional file 1: Table S1** Geographic coordinates (latitude and longitude), sampling date, and environmental radiation of the locations included in this study. **Figure S1** Total dose rates of adult males of the Eastern tree frog (*Hyla orientalis*) collected in areas of medium-high radiation (CEZ-High), and low radiation (CEZ-low) within the Chernobyl Exclusion Zone, and areas outside the Chernobyl Exclusion Zone (Outside CEZ). Box plots represent the interval between first and third quartiles, black lines depict the median value, bars represent minimum and maximum values within 1.5 times interquartile range.

## Data Availability

The datasets generated and/or analyzed during the current study are available in the Figshare repository 10.6084/m9.figshare.14605665.
