# Supplementary material for: Lack of impact of radiation on blood physiology biomarkers of Chernobyl tree frogs
Source: Front Zool. 2021 Jun 29;18:33. doi: 10.1186/s12983-021-00416-x (PMC8240299; doi:10.1186/s12983-021-00416-x)

Supplementary Table S1 Geographic coordinates (latitude and longitude), sampling date, and environmental radiation of the locations included in this study.

Location	Code	GPS coordinates	Sampling date	Radiation (μSv/h)
Azbuchin	AZ	51.4047, 30.1044	15/05/2018	7.61
Vershina	VE	51.4328, 30.0769	18/05/2018	16.20
Dolzhikovo	DO	51.4256, 30.1161	18/05/2018	1.50
Zalesie	ZA	51.2506, 30.1667	17/05/2018	0.12
Lubianka	LU	51.3388, 29.7976	19/05/2018	0.27
Glinka	GL	51.2300, 29.9250	20/05/2018	0.10
Smolin	SM	51.2925, 31.0294	22/05/2018	0.04
Nedanchichy	NE	51.5364, 30.5981	23/05/2018	0.08

Fig. S1 Total dose rates of adult males of the Eastern tree frog (*Hyla orientalis*) collected in areas of medium-high radiation (CEZ-High), and low radiation (CEZ-low) within the Chernobyl Exclusion Zone, and areas outside the Chernobyl Exclusion Zone (Outside CEZ). Box plots represent the interval between first and third quartiles, black lines depict the median value, bars represent minimum and maximum values within 1.5 times interquartile range.

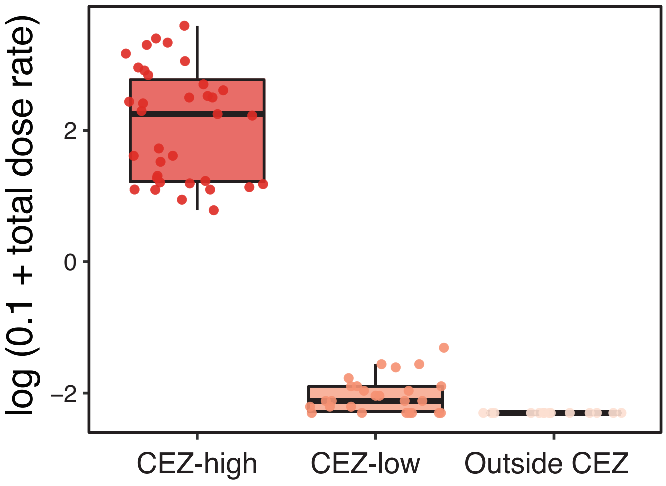

Supplement: Supplementary file 1 — Additional file 1: Table S1 Geographic coordinates (latitude and longitude), sampling date, and environmental radiation of the locations included in this study. Figure S1 Total dose rates of adult males of the Eastern tree frog (Hyla orientalis) collected in areas of medium-high radiation (CEZ-High), and low radiation (CEZ-low) within the Chernobyl Exclusion Zone, and areas outside the Chernobyl Exclusion Zone (Outside CEZ). Box plots represent the interval between first and third quartiles, black lines depict the median value, bars represent minimum and maximum values within 1.5 times interquartile range. [file 12983_2021_416_MOESM1_ESM.pdf]
